# Applying Pebble-Rotating Game to Enhance the Robustness of DHTs

**DOI:** 10.1371/journal.pone.0065460

**Published:** 2013-06-11

**Authors:** LiYong Ren, XiaoWen Nie, YuChi Dong

**Affiliations:** School of Computer Science & Engineering, University of Electronic Science and Technology of China, ChengDu, China; University of Warwick, United Kingdom

## Abstract

Distributed hash tables (DHTs) are usually used in the open networking environment, where they are vulnerable to Sybil attacks. Pebble-Rotating Game (PRG) mixes the nodes of the honest and the adversarial randomly, and can resist the Sybil attack efficiently. However, the adversary may have some tricks to corrupt the rule of PRG. This paper proposes a set of mechanisms to make the rule of PRG be obliged to obey. A new joining node must ask the Certificate Authority (CA) for its signature and certificate, which records the complete process on how a node joins the network and obtains the legitimacy of the node. Then, to prevent the adversary from accumulating identifiers, any node can make use of the latest certificate to judge whether one identifier is expired with the help of the replacement property of RPG. This paper analyzes in details the number of expired certificates which are needed to store in every node, and gives asymptotic solution of this problem. The analysis and simulations show that the mean number of the certificates stored in each node are 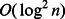, where *n* is the size of the network.

## Introduction

Due to its high scalability, DHT [1]–[4] has won wide attention. But for its actual deployment, scalability is just one aspect of concerns, and security is another problem that cannot be avoided.

The traditional security mainly focuses on such fields as information integrity, tamper-resistance, and nonrepudiation, while things may be a little different in DHT. Generally, the DHT algorithm itself has some redundancy, and random attacks on DHT will not cause great damage. However, if the adversary can gain a great quantity of IDs and use them to start so-called Sybil attack, the redundancy of DHT will not help. Douceur [5] argues that the fundamental issue of security in DHT is how to distinguish different entities. To achieve this, we should either ensure each node can be verified directly, or build a certification authority (CA) to confirm the identities of nodes. Because the method of directly verifying nodes has poor scalability, an explicit or an implicit CA is often used in DHT. For example, the node ID is the hash value of the IP address in CFS [6], while EMBASSY [7] takes advantage of the encryption key of hardware.

Sybil attack poses a dilemma for the design of DHT: either preserving the openness to allow newcomers can easily join the network, or raising the entry barrier to enhance the security of system. Does there exist a tradeoff between the two choices? In this paper, we argue that Pebble-Rotating Game [8] or Cuckoo rule [9] proposed by Scheideler and Awerbuch may be helpful.

PRG is such a game: place black and white pebbles in a ring, white pebble for honest nodes, black pebble for attacking nodes. Assuming that all black pebbles are controlled by the adversary, she has two choices: either inserting a new black pebble into the ring, or removing any black pebble out of the ring. In this game, to insert a pebble into the ring, each participant must follow the *K-rotation* rules: in the first round, the participant randomly replaces a new pebble with an existing one. The replaced pebble then randomly replaces another one in the ring in the second round, …, until the Kth round, the pebble which is replaced in the previous round is randomly inserted into the ring.

Scheideler [8] proves that: in PRG, when 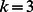, if the adversary could control no more than 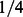 of the total pebbles, then in any continuous sequence of 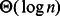 pebbles, white pebbles will win a majority over the black with high probability. When the network size is very large, the premise of this conditional statement is easy to satisfy. But in the practical application of the PRG algorithm, the adversary may try every means to interrupt the *K-rotation* rules by placing the attacking node to the position as she wishes. So there must be some ways to confirm the *K-rotation* rules to be carried out. To solve this problem, we propose an explicit CA to authenticate the K-rotation process, which guarantees that ID can not be forged and the legality of a node can be verified without CA's participation. Furthermore, to prevent the adversary from accumulating IDs, we use recent certificates to judge whether an ID has expired or not.

## Materials

Security needs to be considered at the beginning of the DHT algorithm's design. Castro [10] devides the security of DHT into four areas: security for allocation of node ID, security for maintaining the routing table, security for message forwarding, and security for data. Among them, security for allocation of node ID is the foundation of others. To protect the security of DHT, Castro suggests that the node should pay for its ID or bind the ID to the identity in the real world, but this may limit the applications of DHT.

Sybil attack was first defined by Douceur [5]. As Douceur argues, if we cannot distinguish the identity of a remote node through an explicit or implicit CA, a large part of P2P system will be mastered by the adversary. Furthermore, for a system which relies on an implicit CA, we must be soberly aware how much security such an implicit CA can provide. For example, in IPv6 network, the adversary can easily aquire a lot of addresses, so an implicit CA which is bound to the IPv6 address can not provide sufficient security.

Yu et al. [11] proposes an algorithm called SybilGuard which makes use of the in-and-out degree of social relationship in the real world to distinguish the identity of a remote user. SybilGuard believes there exists a small cut between honest and adversary nodes, and Random-Walk algorithm is used to find this cut. SybilGuard algorithm is suitable for unstructured P2P networks.

In DHT algorithm, before joining DHT network, a node must know an online node in advance and then enter the network with its introduction. According to this bootstrap relations, nodes in the network make up a bootstrap tree. The closer is a node to the root, the higher probability is it to be honest. Danezis [12] takes advantage of this phenomenon and presents some methods to resist Sybil attack, which is more suitable to the hierarchical DHT network.

The consideration of Bazi et al. [13] is more fundamental. He studies how to distinguish the remote entities with network measurement. The lighthouses measure the remote entities, and there exists a lower limit in the measurement values according to the “triangle inequality”. Unfortunately this algorithm is still under development.

Rowaihy [14] tries to build a hierarchical adaptive node management system in the DHT network. In this system, if one leaf node wants to be prompted as a management node, i.e., the internal node of the tree, it must answer puzzles of all its child nodes. These puzzles will occupy a lot of computing resources, which increases the difficulty for the adversary to control other nodes. But at the same time it will waste a lot of computing resources.

Fireflies [15] is an interesting design. In Fireflies, network topology is made up with 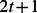 Chord rings, and each physical node joins 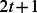 networks simultaneously. The 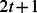 precedings of one node make up the arbitor set of this node, and accusation/rebuttal mechanism is introduced to regulate the behaviors of nodes.

Condie [16] presents a solution similar to PRG and Cuckoo rules. The algorithm adopts a random number generating server as CA, which generates random numbers periodically. The ID of every node is the function of a random number. After every period of time, node IDs will change. Therefore, the locations of the nodes randomly change. Since the node ID can not be predicted from the random number, the adversary can neither pre-select an ID nor accumulate IDs, so the honest and attacking nodes are uniformly mixed. The disadvantage of this solution is that it's not suitable in storage circumstance because the network is changed frequently.

Similar to PRG [8], Cuckoo [9] is designed with replacement operation. When a new node is joining, it will replace all nodes within the distance of 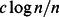. The replaced nodes then will be re-inserted into the network. Awerbuch and Scheideler have proved that under Cuckoo rules, the honest and the attacking nodes are evenly mixed with high probability. Compared to PRG, Cuckoo rules also have the advantage of load balancing. We argue that the load balancing of DHT and Sybil attacks are two orthogonal issues, and other methods can be used to compensate the load balancing in PRG.

Based on PRG, Fiat [17] has imported the Byzantine protocol to handle the cheating of the adversary. Fiat can not only solve Sybil attack, but also is able to handle routing security and data security issues. However, the cost of this proposal is expensive. If it may be tolerable to afford the cost when nodes join the network, it is not affordable for every message to be verified by the arbitor set.

Similar to the design of Fiat, King [18] proposes a security algorithm based on Byzantine protocol and leader selection protocol. However, the cost limits its application.

## Methods

Without loss of generality, we choose Chord [1] as the DHT model in this paper, and apply PRG on it.

### ID generating scheme

To defend the Sybil attack, the first problem to handle is to provide a secure ID generation scheme. If the adversary is able to select any desired ID, then she can put the attacking node to the position as she wishes. So we must deprive her ability to select position in address space, or limit the address space from which she can select. The PRG algorithm makes all nodes to be mixed randomly, which eventually limits the space the adversary can select.

A secure ID generation scheme has several meanings. Firstly, the ID can not be forged. This is easy to understand. If the adversary can forge ID, which means she is able to arbitrarily select a random position, it will undermine the foundation of DHT security. Secondly, the ID should be verifiable, and it is actually another aspect of the former. For one node, how can we determine whether its ID is forged? We argue that the ID should be self-verified. Finally, the ID applied by the node should be constantly changing. If one node always receives the same ID, then the adversary can collect a lot of IDs by different identities, and choose one of them to put the attacking node to the position which she prefers. For example, the adversary can use different IPv6 addresses to register a lot of IDs, then selects a preferred position, which is called adaptive joining attack [9]. So we should restrict the adversary to select an ID in a constantly changing set.

Our ID generation scheme is to make use of an explicit CA. Assuming that every node has a pair of keys 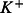 and 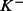, 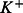 represents the public key, and 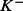 represents the private key. We define the cryptographic operation as 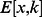, in which the plaintext 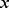 is encrypted into ciphertext by the key 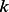. When a node 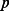 joins the network, it must apply a signature from the CA 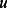. The signature is defined as 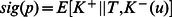, where 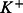 is the public key of 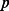, 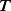 is a timestamp, 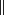 represents the string concatenation operator, and 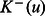 is the private key of the CA 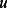. The node ID is defined as the hash value of signature: 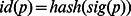.

In this scheme, unless the private key of CA is intercepted, it is difficult to forge a node ID. All nodes can acquire 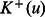, i.e., the public key of CA 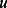, with some methods of out-of-band or Key exchange protocol(IKE). When a node 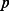 shows its signature, other nodes first use the public key 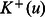 of CA to verify the signature and then use the hash function to calculate the node ID. Finally, since the signature contains a timestamp, it will make the current signature of one node different from the past. So the adversary can not predicate the change of signature and ID.

### K-rotation joining algorithm

Based on secure ID generating scheme, we will apply the PRG algorithm in this subsection. The key to applying PRG is to enforce the K-rotation(Unless otherwise noted, we always assume 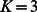 in this paper) rules. Byzantine protocol can handle it well, but due to its cost, we propose to use an explicit CA to carry out the K-rotation rules. When a node joins, CA not only provides the signature to the node but also grants a certificate to its K-rotation joining process. The certification records a complete K-rotation process. Only the signature and certification are legal, can the neighbors of the joining node accept it.

Assuming the process of K-rotation is as follows: in the first round, the new node 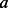 substitutes its direct successor 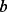. In the second round, node 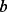 is granted to a new position 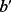, and 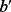 will replace its direct successor 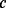. In the third round, the node 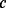 is granted to a new position 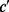, and directly inserted into the new position 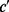. The certification of the former K-rotation is represented as below:
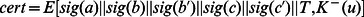
(1)where 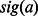 is the signature of 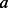, 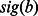 is the old signature of node 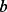, 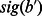 is the new signature of node 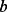, and T is the timestamp when the certification is created. Meanwhile, we define the neighbour of node in address space as 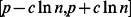, where 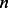 represents the total number of online nodes. With this preparation, we describe the K-rotation joining algorithm as follows:

**Algorithm 1** the K-rotation Joining Algorithm.

Let 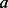 be a new node, 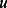 be CA.

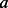
 swaps public key with 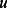, and requests to join the network;

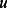
 calculates the signature and ID of 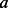, then finds the direct successor of 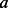;

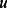
 swaps public key with 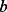, and calculates the new signature of 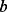 and new ID number 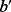;

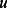
 finds the direct successor of 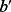;

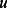
 swaps public key with 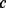, calculates the new signature of 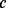 and the ID number 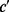 and the K-rotation Joining Replacement certification;

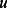
 sends new signature and certification to 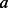, 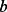 and 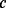;

We assume that the communication between nodes firstly needs to exchange their public keys through IKE. The hash function is used to digest the content of subsequent messages, and the digest encrypted by the private key is pegging back in messages.

Algorithm 0 follows the K-rotation rule. After the Algorithm 0, the nodes (a, b, c) gain new signatures and certifications. Neither old positions nor new positions of the nodes 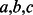 can be predicted.

In the step 3 and 5 of algorithm 0, if the node 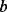 or 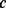 refuses to interact with the CA, i.e. 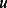, CA will regard 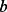 or 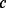 as offline node, and let the next successor be the direct successor. With the help of the ID validation algorithm discussed later, the refusing nodes will be treated as illegal, and the remaining nodes will deport the refusing node from the network.

The steps which are vulnerable to be attacked are step 2 and 4, in which CA may probably not find the proper successor. To prevent this, CA can continuously ask the neighbors near 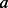 or 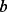 to verify the statements about successor. If CA finds some node lies, it can publish a new certificate to kick out the lying node.

### ID Validating Algorithm

After running Algorithm 0, the new joining node and replaced nodes gain new signatures and certifications. By now these nodes do not really appear in the network topology. Only after the Stabilize Protocol of Chord is run, can other nodes in the network set up connections with them.

Although the adversary can not forge signature and ID, she may still accumulate a lot of signatures and certifications with just one attacking node from the time being. Most of these accumulated IDs have been replaced in K-rotation. If the adversary uses these replaced IDs to join the network through the stabilize protocol, she can violate the K-rotation rules. So we must find a method to patch this hole.

If the neighbour nodes save all previous certifications, when the attacking node tries to join the network again by using the replaced ID, the neighbour nodes then can see through the adversary's attempts after looking up saved certifications, and patch this hole. But it is unrealistic to completely save all old certifications.Do there other ways exist, which can significantly reduce the number of certification needed to save?

Notice that in algorithm 0, the node replaced by node 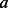 is its direct successor 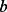, so there are no online nodes existing in range 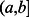. Similarly, there are no online nodes existing in range 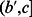. 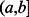 and 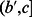 are called as replacement intervals in this paper. When the adversary wants to let a node 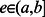 join the network, then the node 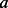 and its neighbour nodes can use 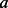
's saved certification to deduce whether 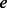 is an expired ID and refuse the joining request of 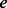.

Based on this idea, in this paper, neighbour nodes save recent certifications to verify whether an ID is expired. The next problem is how many certifications need to be saved in the neighbour nodes? If the replacement intervals of recent certifications can completely cover the neighbour area 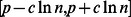, then the older certifications are not necessary to be saved. Thus the minimum number of recent certifications saved by neighbour is to cover the complete neighbour range.

On each node, a database 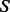 is set up to save all recent certifications within neighbour range, and it is required that the replacement intervals of the recent certifications can completely cover the neighbour range. The algorithm 0 is running on nodes to verify whether an ID is expired.

**Algorithm 2** ID Validation algorithm.

Let 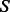 be the certification database, 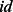 be the node needed to be verified, 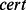 is the certification of 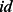.

1. If 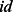 is not in the neighbour range, return illegal;
2. If 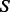 contains a certification newer than 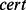, and 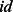 is fallen into the replacement interval of the certification, then the 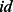 is illegal;
3. Otherwise, accept 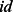 as legal, and insert 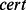 into 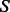.

In algorithm 0, first we need to check whether the id is within the neighbour range. If it is not, we mark it as an illegal 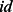. If there is a certification in database 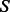 which covers this 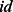, and the certification timestamp is newer than 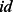, then mark this 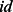 illegal. The logical branch of step 3 in algorithm 0 contains: (1) 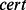 is newer than any other certifications in 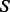; (2) 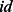 is not covered by 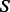. The logical branch (1) corresponds to the new certification, while (2) is probably because the certifications in database 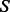 can not completely cover the whole neighbour range.

Each node exchanges the database 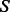 periodically with its neighbours. If the timestamp of received certification is newer, the node inserts the certification into its local database 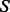. If the replacement interval of the new inserted certification covers the replacement interval of the old ones, just delete the old one. Indeed, if one of the neighbour nodes is honest, the complete certification database can be inherited and propagated.

By using the ID validating algorithm with the Stabilize Protocol, we can verify whether a node is legal or not. We just neglect the request of illegal nodes, then the illegal nodes will be kicked out of the network.

## Discussion

In algorithm 0, we use the recent certifications saved in database 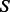 to verify whether an ID is legal. It is required that the replacement intervals of all certifications should cover the neighbour range. Compared with the method of storing all certifications, the certification number in algorithm 0 is much less. However, if the number of certifications needed to save is still large, then the applying scheme of PRG proposed in this paper is still unpractical. In this section, we will analyze this problem.

We use another presentation to describe the considered problem: how many certifications are needed to completely cover the network address space? If the replacement interval is treated as segments, the problem is converted into a circle covering problem. Feller [19] has made an excellent summary on how to use equal-length segments to cover a circle. But not completely in accordance with what Feller describes, the length of covering segments is variable. Fortunately, there are many mathematicians trying to solve how to use random-length segments to cover a circle. Siegel [20] and Domb [21] use different methods to solve this problem. In this paper, we use Domb's method.

First we give Domb's conclusion. Assuming 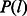 is the probability for a circular arc with length 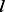 to be covered, we define the initial probability as 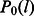, which represents the probability of that the starting point of a covering segment is fallen ahead one circle arc and the arc is covered. Meanwhile we introduce the intermediate function 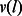, which is defined as (7). And let the PDF of covering segments be 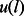. According to Domb [21], the probability that the circle is covered by random segments is:
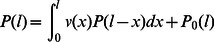
(2)


Since 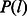 in (2) is a convolution, we then transform formula (2) by using Laplace’s transformation and we get:
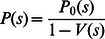
(3)


In the following, we will apply formula(2) and formula(3) to solve our problem. First we introduce two lemmas. Limited to the length of paragraph, we omit the proof of them.

### Two Lemmas

In a unit circle whose circumference length is 1, we randomly insert 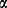 nodes. The probability of the number of nodes that appears on one arc and the interval between nodes obey the following distributions:

**lemma 1** *If nodes randomly select ID in address space, then the node interval obeys negative exponential distribution, 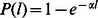.*

**lemma 2** *If nodes randomly select ID in address space, then the number of nodes appearing in an arc obeys the Poisson distribution, 
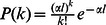
.*

According to lemma 1, the length of the replacement interval is a random variable, which obeys the negative exponential distribution with parameter 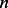, where 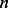 is the network size. Thus the PDF and CDF of the replacement interval are respectively 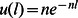 and 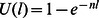. Let the number of total covering segments be 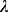, and the clockwise direction be positive. According to lemma 1, the starting location of every covering segment obeys the negative exponential distribution with parameter 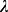.

### The Initial Distribution

By lemma 2, we get the initial distribution 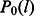:
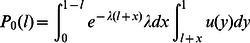
(4)


In formula(4), 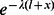 represents that there are no starting points of covering segments in 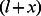, and 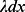 represents there is one starting point of covering segments appearing on the differential element 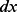, while 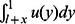 represents all covering segments whose length exceeds 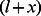. Since the covering segments distribute in the interval 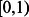, the integral limit of 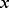 is from 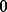 to 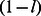. The formula (4) can be reduced as:
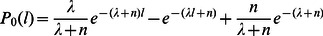
(5)


According to formula(5), when 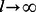, 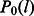 converges to 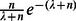. Using Laplace's transformation on 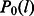 with 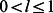, we get:
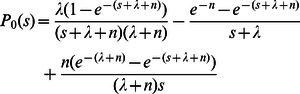
(6)


### Intermediate Function

According to Domb [21], the intermediate function 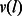 is defined as:
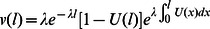
(7)


Substitute it into the CDF 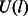 of covering segments:
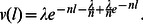
(8)


Make Laplace's transformation on 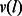


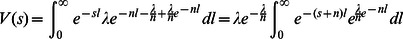
(9)


According to formula (9), 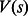 exists. Since the solution of 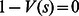 is the pole of (3), and the position of pole can directly affect the time-domain properties, we must have detailed understanding of 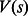.

Limited to the length of this paper, we omit the detailed analysis on 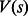. After analysis, 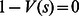 only has solutions in the real axis. While 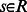, 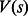 is a monotone decreasing function. 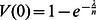, 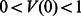; When 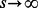, then 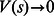; Thus the solution of 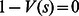 is on negative real axis. Assume the solution is 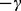.

### Covering Probability

Plugging the formula (6) and (9) into formula (3), we get:
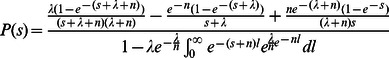
(10)


In case of the complex form of formula(10), here we just consider its asymptotic solution. For a Laplace transformation, its pole position decides the time-domain transition process. In formula (10), there are four possible positions for pole: 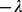, 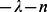, 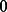, 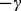. Since 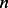 is the network size, 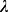 is the number of covering segments, thus 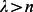. In the formula (10), the coefficients of pole 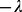 and 0 are both very little and these two poles can be neglected. Thus:
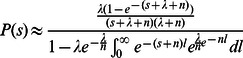
(11)


In order to further solute the asymptotic value of (11), using Domb's conclusion: when 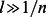, where 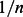 is the average value of covering segments, 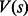 is asymptotically equal to the intermediate function by using equal segments 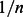 to cover the circle. This asymptotic intermediate function is [21]:
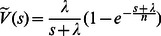
(12)


When 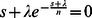, where 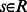, let the solution be 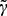, where 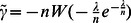, and 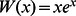 is the Lambert-W function [22].
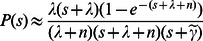
(13)


Expand the formula (13), and we get:
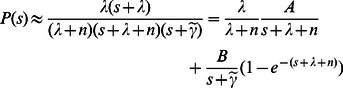
(14)

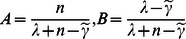



Make Laplace Inversion Transformation on formula (14), and we get:
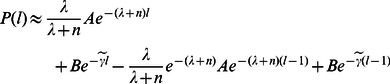
(15)


In the above formula, when 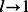, all others items can be ignored. Thus:
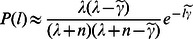
(16)


Formula (17) is the probability when 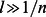 and the circle with length *1* is covered. The problem this paper needs to consider is the probability when the unit circle is covered, thus 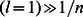. And in algorithm 0, every time when a new node joins the network, a certification will be generated, and each certification contains two replacement intervals, thus 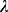 should be multiplied by 2. If let 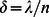, we finally get:
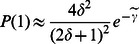
(17)where 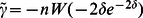


When the probability of formula(17) is 0.5, 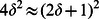. Solving the formula 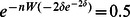, then we get:
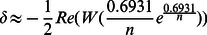
(18)where 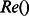 represents getting the real part, and the Lambert-W function gets the 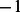 side. Since 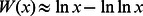 [22], we get:



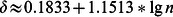
(19)Because every node needs to save the certifications of neighbours, according to the formular (19), we have the following theorem:

**theorem 3** *In the scheme of this paper, the certification number saved on nodes asymptotically is* 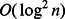.

**Figure 1:**
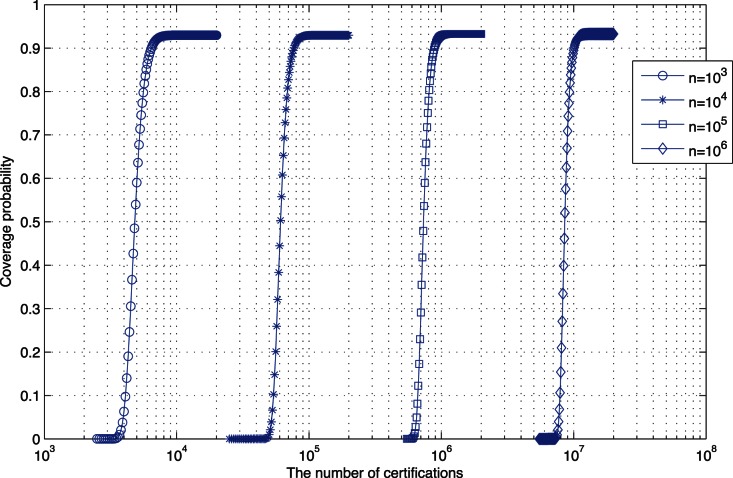
Under different network size, the probability changes with the increasing of 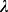. By formula (17), where 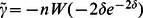, 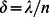, the Coverage Probability changes with the increasing of 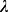 under different network size.

By formula (17), when 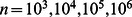, the probability with 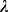 changes, as shown in Figure 1. In Figure 1, we can observe that 1) The 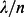 value of every curve is not large. When 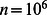, the 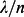 value is the largest, a little larger than 10; while 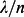 represents the average number of certifications in every node. 2) In a network with size 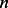, the probability to completely cover the address space increases to 0.9 in the narrow 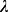, which indicates that the capacity of the certification database 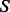 is changing in a narrow range. 3) The intervals of these four curves are almost the same in logarithmic coordinate, which indicates that with the network-size 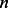 increasing, the increasing amount of 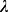 is almost proportion to 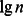. By (19), it is well explained.

With the above analysis, the average capacity of certification database 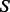 is not large. For a certain network size, 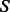 is changing in a very narrow range. Considering that every node should keep the certifications of neighbour nodes, the capacity of 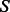 is 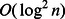.

## Results

We use simulation tests to verify our analysis in this section. In the simulations, we select 32-bit unsigned number as the ID space, and use SHA1 algorithm to generate the random number. At the beginning of the simulation, first we generate 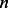 numbers of ID and let them join the network. After an experiment begins, we keep generating nodes and let them join the network, and we randomly choose an online node to make it leave the network, until all space is covered.

Some caution is needed to be taken here. At the time to select a random node to exit the network, we can not first generate a random ID and let the successor of the ID leave. The nodes chosen like this can not guarantee the uniform distribution [23].

From the results of 5000-time experiments, the range of 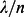 is within 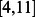, and this range is not large, which indicates that in a network with 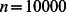, the average number of certification (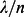) to cover the address space is within range 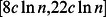. The cost is acceptable.

### The Distribution of 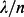


**Figure 2:**
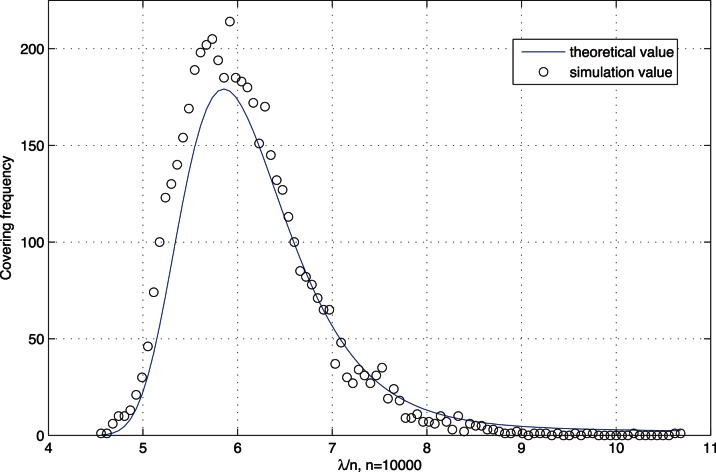
With network size *n*  =  10000, the probability distribution to cover the address space. The theoretical probability distribution is calculated by formula (17) which is represented as a solid line, and the simulation result is pointed by little circles.

In this test, we try to verify whether the analysis on the probability distribution is correct. To do this, we count the frequency of the certifications 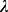 to completely cover the whole address space. Select network size 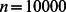, and take 5000 rounds tests and then collect the distribution condition of 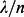. The test result is shown in Figure 2. The horizontal cordinate is 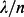, and the vertical coordinate is frequency.

In Figure 2, the theoretical probability distribution is calculated by (17) which is represented as a solid line, and the simulation result is pointed by little circles. Observing the Figure 2, we can see that the simulation result and the theoretical result match well, which indicates that although the distribution function of 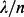 is asymptotic, the asymptotic loss is little. So it is acceptable to make use of (17) to caculate the mean value 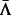, i.e., the capacity of the certification database.

### The Number of Certifications in Different Network Size

In this subsection, we test the number of certifications to cover the whole address space in different network size. We use different network size to do the tests, from 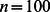 to 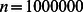. For each network size, we take 100 times tests.

**Figure 3:**
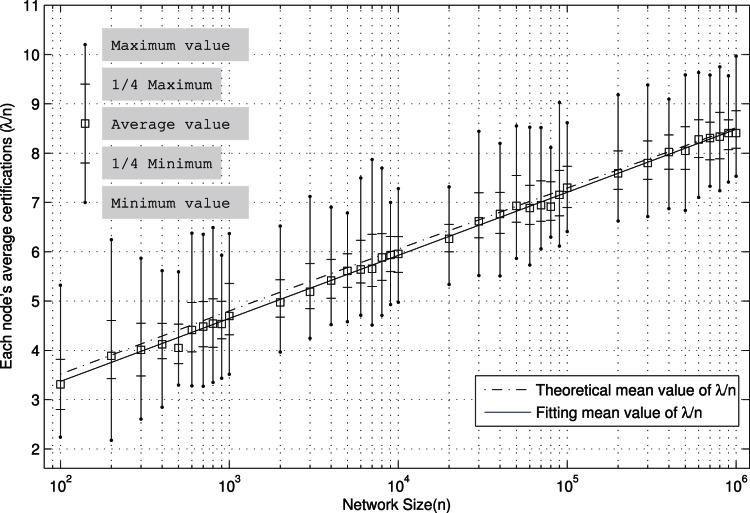
In different network size, the average number of certifications 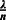 every node saved. The horizontal coordinate represents the network size, and the vertical coordinate is the average number of certifications saved on every node (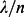). For each network size 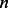, the mean value, 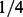 maximum value, and 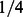 minimum value of 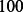 rounds tests are calculated.

The test results are shown in Figure 3. The horizontal coordinate represents the network size, while the vertical coordinate is the average number of certifications saved on every node (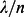). For each size 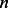, we calculate the mean value, 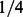 maximum value, and 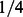 minimum value of 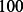 rounds tests.

Observing the Figure 3, with the increase of network size, the average number of certifications to save on each node is increasing too. And the average value of 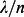 shows to be a linear growth under the logarithmic coordinate 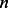. We fit the average curve of 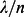 with the least squares.
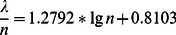
(20)


The linear fitting on 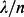 is very close to the mean value. Compared (19) with (20), we can find that the difference between the coefficient of 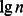 is quite small. And in Figure 3, the theoretical mean curve calculated by (19) is very similar to the curve by (20).

### Conclusions

In an open DHT network, it is difficult to defend the Sybil attack. The PRG algorithm requires that the number of nodes under the adversary's control can not exceed 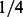 of totals. This condition can be easily satisfied when the DHT network size is very large. But how to carry out the K-rotation rules without being interrupted is a barrier to apply PRG.

Since the cost of the Byzantine algorithm is expensive, we propose to use an explicit CA to handle this problem. CA is used to send signatures and certifications to the joining nodes, which can guarantee that ID is verifiable and can not be forged. The certification records a complete K-rotation joining process, and it is the credential of nodes to enter the network. In order to prevent the adversary from using the expired IDs to join the network by Stabilize Protocol, we propose to make use of the replacement intervals of certifications to verify whether an ID is overdue. This ID validating algorithm requires that the saved certifications can completely cover the whole neighbour range, and the key problem is how many certifications are suitable. In this paper, we convert this key problem to covering a circle by random segments. We analyze the covering problem in details, and derive an asymptotic solution to it. The analysis and simulations show that the average number of certifications to be saved on each node is small, which is 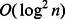, and indicates the scheme proposed in this paper is applicable.
